# Explainable AI in breast cancer ultrasound imaging: current developments and challenges

**DOI:** 10.3389/fdgth.2026.1846763

**Published:** 2026-06-15

**Authors:** Madiha Hameed, Kok Swee Sim

**Affiliations:** Faculty of Engineering and Technology, Multimedia University, Bukit Beruang, Melaka, Malaysia

**Keywords:** Explainable Artificial Intelligence (XAI), breast cancer, ultrasound imaging, deep learning, Grad-CAM

## Abstract

Breast cancer has been one of the most common causes of cancer mortality in the world, and thus, early and correct diagnosis is crucial in enhancing patient outcomes. The use of ultrasound imaging as a complementary diagnostic tool is very common because it is safe, accessible, and cost-effective, especially in resource-strained environments. The deep learning methods have achieved spectacular success in the past few years in their effort to automate the process of detecting and classifying breast cancer based on ultrasound images. The transparency of these models, however, is not always clear, and this problem is commonly known as the black-box problem, which is a serious obstacle to the implementation of these models in clinical practice. Explainable Artificial Intelligence (XAI) is an emerging technology that opens business opportunities to improve the interpretability and reliability of deep learning models by offering insights into the decision-making process of these models. This mini review will be a summary of the current developments in XAI applied to the ultrasound image of breast cancer, such as saliency-based approaches, model-agnostic methods, and attention mechanisms. Moreover, it outlines some of the most significant obstacles, which include a lack of standardized evaluation metrics, clinical validation, and the fact that it is hard to interpret an explanation of noisy imaging conditions. Lastly, possible future pathways are outlined to close the gap between the currently successful AI systems and their successful application to clinical practice.

## 1 Introduction

Breast cancer is considered to be one of the most common cancers and one of the leading causes of death amongst women all over the world. Early diagnosis greatly enhances the survival rates and minimizes the complexity of the treatment, and thus, proper diagnosis at the right time is an important aspect of a good health care system. Ultrasound imaging, among other imaging modalities, has become very significant as it is non-invasive, devoid of ionizing radiation, and is cheap (1–3). It has specific beneficial uses, specifically in screening dense breast tissues, and is commonly employed in developing countries where more and more advanced imaging technologies may be of limited accessibility (4). Nevertheless, the interpretation of ultrasound images is very operator sensitive and may be prone to variation, thereby impacting the quality of the diagnosis. Over the past few years, computer-aided diagnosis systems have been transformed due to the introduction of deep learning methods into the medical imaging field (5). Convolutional Neural Networks (CNNs) and, more recently, Vision Transformers (ViTs) have shown impressive results in vision tasks like classification of tumours, lesion detection, and segmentation in breast ultrasound images (6). In spite of these developments, one of the largest weaknesses of such models is that they are not interpretable. These systems are usually black boxes, they offer predictions, but it is not clear how they arrived at the prediction, and this brings up issues of reliability, accountability, and clinical acceptability (7). To overcome this shortcoming, Explainable Artificial Intelligence (XAI) has become a highly important field of study that is directed at enhancing the interpretability of machine learning models. Gradient-weighted Class Activation Mapping (Grad-CAM), Local Interpretable Model-agnostic Explanations (LIME), and SHapley Additive exPlanations (SHAP) are XAI methods, which will offer visual or feature-wise explanations to clinicians about how decisions are made (8). XAI can improve the levels of trust, clinical decision-making, and the implementation of AI-based tools into the real medical environment in the context of breast cancer ultrasound imaging (9). This mini review will elucidate a narrow scope of the current trends in explainable AI in breast cancer ultrasound imaging. It covers major methodologies, mentions the prevalent challenges, and some of the gaps and the direction of future research, which can be used to establish credible and interpretative diagnostic systems. The following are the main contributions of this mini review:

1. **Detailed description of XAI in the Breast Ultrasound Imaging:** It is a brief but systematic overview of current developments in explainable artificial intelligence methods that have been used in breast cancer detection based on ultrasound imaging.
2. **Explainability Methodology Categorization:** Current XAI methods are systematically divided into saliency-based, model-agnostic, and attention-based methods, which allow for a clearer picture of how they work and where they are used.
3. **Critical Review of the Current Methods:** The review gives strengths and limitations of the existing XAI techniques, especially in noisy and low-contrast ultrasound imaging.
4. **Research Gap Identification:** The challenges that could be identified and discussed include the absence of standardized measurement of evaluation, the issue of limited clinical validation, and variation in the reliability of explanations.
5. **Future Research Directions:** There are identified potential directions in which the work can be done in the future, such as building hybrid explainable models, better interpretability methods, and incorporating them into clinical decision-support systems.

**Figure 1:**
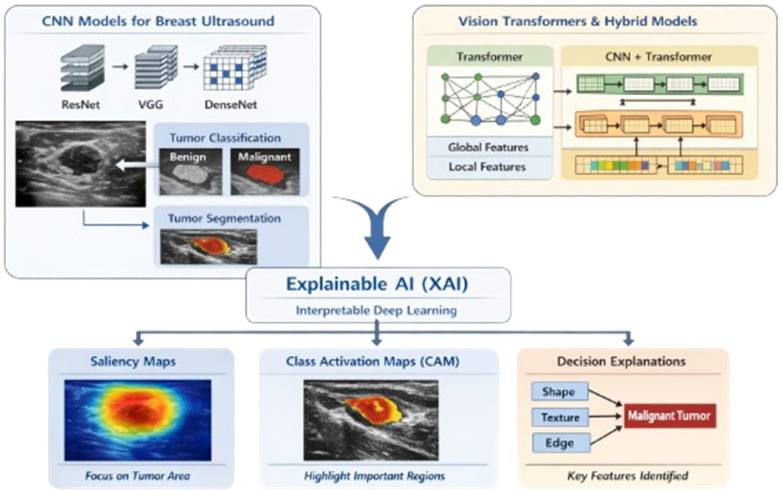
Explainable AI and deep learning application in breast cancer detection.

Figure 1 demonstrates the deep learning workflow of breast ultrasound imaging, featuring the succession of CNN-based architectures, including ResNet, VGG, and DenseNet, and Vision Transformers and hybrid architectures, combining both local and global feature extraction. It notes the role of these sophisticated frameworks in improving the classification and segmentation of breast cancer tasks, besides noting the incorporation of Explainable AI (XAI) methods, such as saliency maps and class activation maps, to increase interpretability. All in all, the figure presents that deep learning, along with XAI can bring more transparent, reliable, and clinically meaningful predictions in breast cancer diagnosis.

## 2 Deep learning in breast ultrasound image

Medical imaging has been transformed by deep learning, as it is now possible to extract features and make high-accuracy predictions without manually-crafted features. CNN-based architectures such as ResNet, VGG, and DenseNet have seen widespread applications to the problem of breast cancer classification, detection, and segmentation in ultrasound imaging (10). Such models are especially efficient in the learning of hierarchical spatial characteristics, providing the possibility to differentiate benign and malignant lesions with high accuracy. Also, encoder-decoder models such as U-Net and its variations have demonstrated exceptional results in tumor segmentation by maintaining minute spatial detailing (11). To improve this even further, the concept of transfer learning has become very popular in research, with the intensive models using large-scale datasets such as ImageNet being fine-tuned on the datasets of breast ultrasound (12). The methodology assists in overcoming the problem of a lack of annotated medical data and enhances the generalization of the model (13). In addition, data augmentation methods, such as rotation, flipping, and noise injection, are widely used to enhance the diversity of the dataset and minimize overfitting ViTs have recently acquired considerable popularity because they can capture contextual information on a global scale, although they have not focused on modeling long-range dependencies (14). Transformers, unlike CNNs, consider the whole image when learning relationships by use of self-attention mechanisms (15). Architectures that integrate CNNs with transformers have also been suggested to take advantage of local feature extractions and global context modeling, and produce better diagnostic results in complex ultrasound images. Moreover, more sophisticated deep learning models like attention mechanisms, multi-scale feature fusion and ensemble learning have been proposed to further enhance model accuracy and robustness (16). Attention modules, such as, allow the network to concentrate on clinically relevant areas, and it enhances the localization of lesions and interpretability to a certain degree. These achievements notwithstanding, deep learning models are described as black-box systems because they are not transparent and interpretable. It is also a severe hindrance to clinical adoption because medical personnel need clear explanations to rely on automated decisions. As a result, interest in incorporating the Explainable Artificial Intelligence (XAI) methods with the deep learning models to offer both visual and quantitative understanding of the model predictions has increased as a way of improving the reliability, accountability, and clinical acceptability (17).

## 3 Breast cancer grad camp visualization

**Figure 2:**
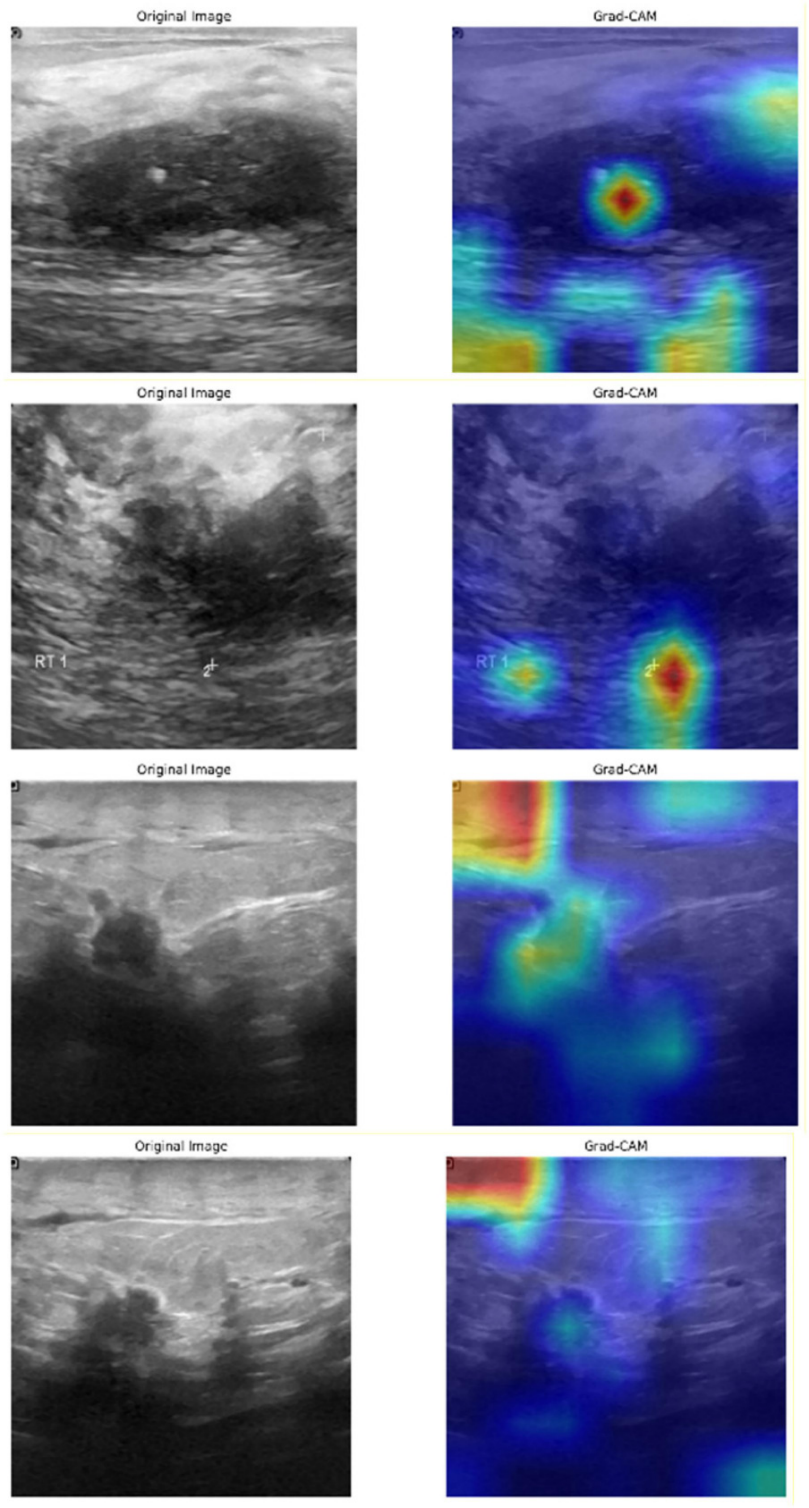
Grad-CAM visualization results for malignant breast ultrasound images showing the regions of interest learned by the deep learning model. The highlighted heatmap areas indicate clinically significant tumor regions and abnormal tissue structures associated with breast cancer.

To understand the predictions of the deep learning model for breast ultrasound images, grad-CAM visualization was used. The heatmaps generated by this model showed that it was able to focus on the clinically relevant regions of the tumor, such as regions of irregular boundaries and abnormal tissue structures, which are considered a characteristic of malignant breast cancer. These visual explanations show the ability of deep learning models to learn meaningful diagnostic features and enhance the transparency of the model and the reliability of computer-aided breast cancer diagnosis systems. Figure 2 presents the results.

## 4 Breast cancer ultrasound applications

**Figure 3:**
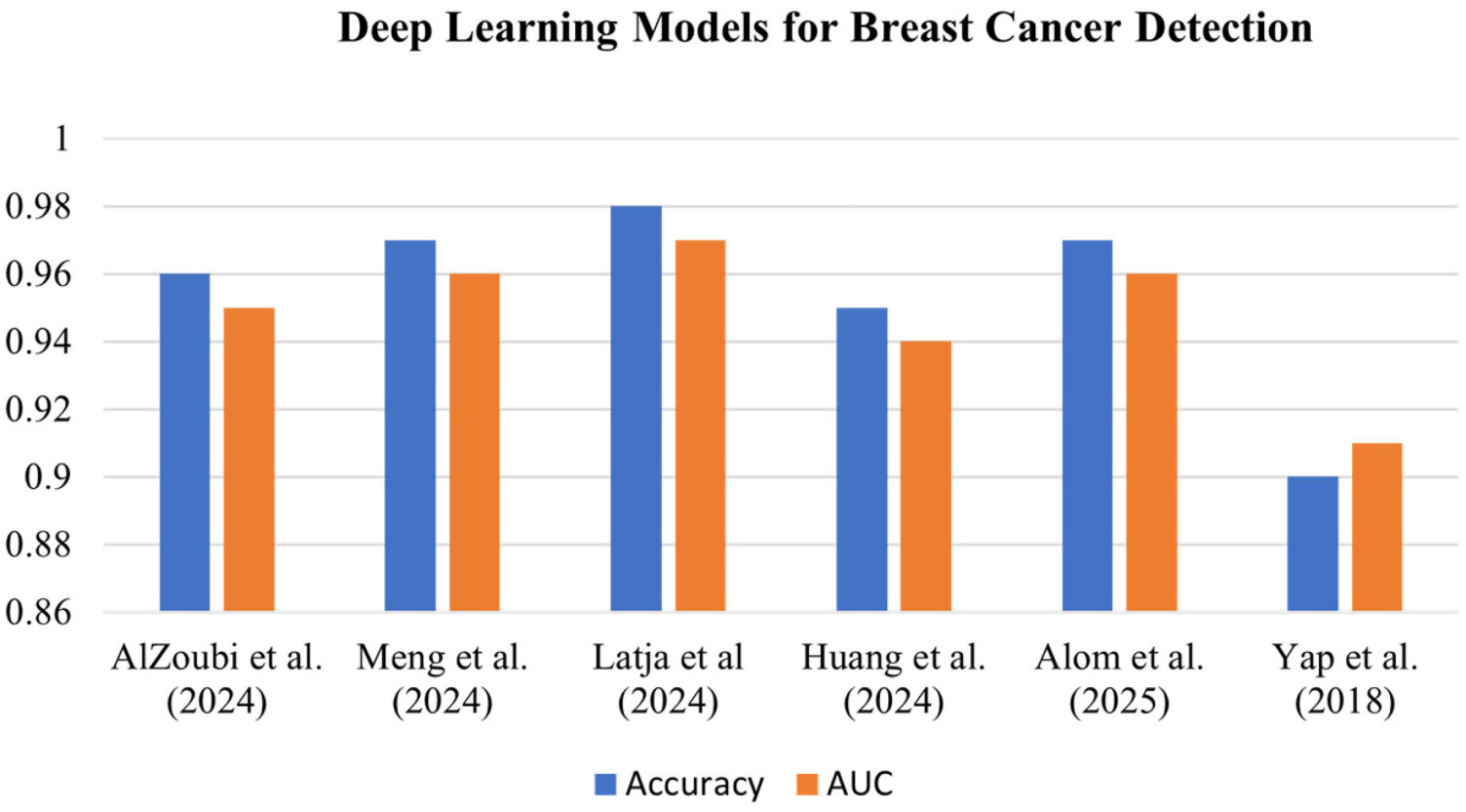
Deep learning models for breast cancer detection.

There has been a growing use of explainable Artificial Intelligence (XAI) techniques in breast ultrasound imaging on a broad range of tasks, such as tumor classification, lesion detection, segmentation, and risk assessment. The role of XAI in breast ultrasound imaging is imperative in assisting the clinician in making sense of model predictions to enhance trust, accountability and clinical adoption. Generally, the XAI techniques can be divided into saliency-based, model-agnostic, attention-based, and emerging concept-based ones. One of the most popular techniques of visual explanation is saliency-based ones. Other techniques like Gradient-weighted Class Activation Mapping (Grad-CAM), Grad-CAM++, and Score-CAM produce heatmaps that show areas in an image that are the most impactful in the prediction made by a model (18). The methods are especially applicable in breast ultrasound, where they may be used to localize suspicious lesions or masses. They are appropriate due to their simplicity, ability to easily integrate into CNN systems, and easy-to-interpret visualizations but can be inaccurate and rough or noisy in their explanations and do not draw fine boundaries. Local Interpretable Model-agnostic Explanations (LIME) and SHapley Additive explanations (SHAP) are model-agnostic methods of explanation, and are not tied to a specific model architecture. LIME estimates the model locally with interpretable surrogate models, whereas SHAP gives contribution scores to each feature via cooperative game theory. These techniques are general and may be used on any machine learning or deep learning model. In medical imaging, they are applicable in the interpretation of feature importance in medical imaging and in justifying the choice of models, but are computationally costly and do not have spatial coherence in the application to image data. Attention-based approaches provide a more holistic, interpretable approach because they incorporate explanatory mechanisms into the model structure (19). However, specifically, ViTs and attention-based CNNs produce attention maps, which are maps showing the regions of the image with which the model concentrates when making predictions (20). These attention maps give a more organized and usually more modeled clarification than *post-hoc* saliency methods. Attention mechanisms utilized in the analysis of breast ultrasounds are used to emphasize diagnostically relevant areas and enhance the performance and interpretability of the findings at the same time (21). These conventional categories are in addition to concept-based explanation techniques like Testing with Concept Activation Vectors (TCAV) which are newly developed (22). These approaches aim to describe model predictions using high-level human-intelligible notions such as shape, margin irregularity or patterns of lesion texture, which are of particular interest in a clinical environment. Moreover, counterfactual explanations offer information and understanding that can be altered concerning the changes that need to be minimal to alter a model prediction and offer useful views on decision validation. In spite of the great development of XAI techniques, there are a number of challenges (23). Most methods are not consistent and robust and thus, they give dissimilar explanations of similar inputs. Moreover, the clinical usefulness of explanations is not necessarily ensured and quantitative analysis of the interpretability is an open research question. Thus, the prospective research ought to consider creating standardized evaluation measures, enhancing the faithfulness of explanations, and creating XAI frameworks that are clinically meaningful and specific to breast ultrasound imaging (24) Table 1 presents the key performance of deep learning models for breast cancer detection, and Figure 3 presents their results and achievements.

**Table 1:** Key performance of deep learning models for breast cancer detection.

References	Method	Dataset used	Key performance
AlZoubi et al. (24)	Explainable DCNN (Grad-CAM)	Private Breast US Dataset	Accuracy = 96%, AUC = 0.95
Meng et al. (21)	CNN + Vision Transformer	BUSI Dataset	Accuracy = 97%, AUC = 0.96
Latja et al. (20)	CNN + Grad-CAM	BUSI Dataset	Accuracy = 98%, AUC = 0.97
Huang et al. (12)	Multi-ask DL (3D Ultrasound + XAI)	3D Breast US Dataset	Accuracy = 95%, AUC = 0.94
Alom et al. (4)	Multi-mo dal DL + XAI	Histopathology + US	Accuracy = 97%, AUC = 0.96
Yap et al. (25)	CNN for lesion detection	BUS Dataset	Accuracy = 90%, AUC = 0.91

## 5 Challenges and limitations

With considerable advances in the process of incorporating Explainable Artificial Intelligence (XAI) into breast ultrasound imaging, a number of essential challenges and limitations to its common clinical use exist. The need to have standard evaluation metrics to evaluate the quality of explanations is one of the main problems (26). In contrast to the classical performance metrics, e.g., accuracy or F1-score, interpretability does not have any quantitatively agreed-upon standards, thus making it hard to compare the performance of various XAI models objectively, as well as to prove their usefulness or not. Reliability and stability of the explanations is another significant issue (27). The XAI methods can often deliver inconsistent or illusory results, i.e., when input data changes slightly, the explanation changes dramatically. In ultrasound imaging, this is a bigger issue because of natural issues like speckle noise, low contrast, and imaging artifacts which may adversely affect model predictions and their explanations. The question of faithfulness is also critical because not all of the methods of explaining the process can show the actual decision-making process of the model (28). As an example, saliency maps could be used to draw attention to areas that look semantically important but that are not relevant in the internal calculations of the model. This may cause erroneous confidence on the side of clinicians, and unsafe clinical choices. Also, lack of clinical validation is still a major obstacle. Numerous studies are done on small datasets that are curated and have not been validated in clinical practice. This means that the usefulness of XAI methods in practice is usually questionable, and their usefulness in helping radiologists has not been determined in large-scale clinical trials. The other challenge that is critical is generalization and robustness. Models that are trained on a particular dataset tend not to be consistent on different institutions, imaging devices or on different patient populations. The difference in ultrasound acquisition protocols and the dependence of its operator also makes this problem more complicated and decreases the accuracy of both the prediction and explanation in different settings. Interpretability does not necessarily mean clinical usefulness, based on a usability viewpoint. Technically, some of the explanations might be correct yet hard to unravel or incorporate into the clinical workflow. This brings about the need of designing XAI systems that are clinically meaningful and user-friendly, but one that suits the needs of healthcare professionals. Further, the computational complexity and scalability are also a problem especially in model-agnostic strategies, such as SHAP, which are resource-sensitive when used in high-dimensional medical images. This may restrict their application in real time clinical environments where efficiency matters a lot. The ethical and regulatory issues are also a factor such as matters of accountability, transparency, and prejudice. When a model is being biased through training data, XAI techniques will reinforce or not sufficiently identify these biases. It is crucial to be just and uphold medical standards thus. All in all, even though XAI has significant potential to enhance the level of transparency and trust in breast ultrasound imaging, the issues mentioned above are vital in its successful implementation in real clinical practice. This should be done in future by ensuring more robust evaluation structures, an increase in the reliability of the explanation, and comprehensive clinical validation to provide safe and effective implementation Table 2.

**Table 2:** Deep learning model’s key contributions and limitations.

Reference	Scope & focus	Key contribution	Dataset used	Limitations
AlZoubi et al. (23)	Classification + XAI	Saliency-based explainable DCNN framework	Retrospective US dataset (1298 images)	Limited generalization; visual explanation only
Meng et al. (24)	CNN + Transformer	Interpretability + combined local/global features	BUSI	Needs larger multi-institutional testing
Madhu et al. (30)	Segmentation/Classification	Hybrid U-Net + Capsule Network model	BUSI	Higher computational complexity
Wang et al. (29)	Explainable segmentation	Transformer-based explainable framework	CBIS-DDSM	Requires more clinical validation
Huang et al. (12)	3D whole breast US interpretability	Multi-task deep learning, molecular expression prediction	3D breast US dataset	Computationally intensive; needs broader validation
Carrilero-Mardones et al. (18)	BI-RADS descriptors from US	DL model learns clinical descriptors	Clinical BUS dataset	Focus on BI-RADS not solely classification
Alom et al. (4)	Explainable cancer detection	Combines histopathology + US with XAI	Multi-modal datasets	Complexity; generalization remains to be validated
He et al. (3)	Hybrid segmentation	CNN-Transformer for US segmentation	Public US datasets	Segmentation focus; interpretability not central

## 6 Future direction and discussion

The research in future must be aimed at coming up with effective, reliable, and clinically significant XAI techniques specifically designed to be used in medical imaging application like breast ultrasound. Although the current methods have been helpful, there is increased pressure to develop methods that will not only describe the model prediction but also be in line with radiological concepts and diagnostic specifications. A promising future is hybrid architecture that integrates CNNs, Vision Transformers, and explainability mechanisms since it can take advantage of the local feature-based extraction mechanism and the global contextual knowledge and provide better interpretability. One of the priorities is the formation of uniform assessment systems of XAI. Future research ought to attempt to establish quantitative and qualitative measures, e.g., faithfulness, localization accuracy, stability, and clinical relevance, to measure the quality of the explanations systematically. Benchmark data sets and common evaluation procedures would also be used to easily compare and replicate different methods. The other direction that is worth considering is the integration of XAI in the real-life clinical workflow. This entails the development of an interface displaying easy to understand visualization interfaces which bring out explanations that are intuitive and actionable to the clinician. The researchers of AI, radiologists, and healthcare professionals will need to cooperate and collaborate to make sure that the results of XAI can be interpreted, relevant, and useful in clinical decision-making. Enhancing explanation in noisy and low-contrast ultrasound images is still a major issue. Further development of noise-resistant designs, models of uncertainty, and sophisticated preprocessing are potential areas of future work to improve the prediction and explanation reliability. The addition of domain knowledge, which can be anatomical priors and lesion properties, can also contribute to the interpretability of the models in difficult imaging cases. Also, there is a promising potential of the research in the future through multi-modal and multi-task learning frameworks. With the integration of ultrasound imaging and clinical data, histopathology, or any other imaging format, models will be able to produce richer and more contextualized explanation. Equally, the use of XAI in the detection, segmentation, and classification of diseases at the same time can offer a more in-depth understanding of the characterization of diseases. The other new avenue is the creation of concept-based and human-centered explanations, trading model choices into clinically relevant concepts like lesion form, margin abnormality and tissue heterogeneity. Such methods can make a substantial improvement on trust and usability as opposed to low pixel pixel-based explanations. Also, interactive XAI systems and counterfactual explanations can allow clinicians to investigate what-if situations, making the decisions made with AI assistance more likely to be understood and trusted. In terms of deployment, scalability, efficiency and applicability of XAI methods in real-time should also be discussed in future research. Computationally efficient and lightweight methods will be required in the implementation to clinical systems and point-of-care equipment. Lastly, regulation compliance and conduct of numerous clinical trials are essential to put XAI-enabled models into practice. The studies must be large-scaled and multi-centered to measure the efficiency, safety of these systems, and their general applicability in a variety of patient populations and imaging conditions. Considering ethics such as bias reduction, transparency, and accountability will also help to make AI use in healthcare responsible and trustworthy. Altogether, the development of XAI in breast ultrasound imaging is a multidisciplinary project that should be based on technical creativity, clinical intuition, and strict validation. Further research can narrow the gap between the experimental models and real-world implementation by overcoming the current limitations and concentrating on the clinically relevant solutions, leading to the ultimate improvement of the diagnostic accuracy and patient outcome.
